# Endoscopic gastric conduit plication: a novel endoscopic technique for delayed gastric conduit emptying following oesophagectomy

**DOI:** 10.1308/rcsann.2025.0025

**Published:** 2026-02-02

**Authors:** H Room, T Bhuvanakrishna, D Peristeri, S Preston, J Kelly

**Affiliations:** ^1^ University Hospital Southampton NHS Foundation Trust, UK; ^2^ East Suffolk and North Essex NHS Foundation Trust, UK; ^3^ Royal Surrey NHS Foundation Trust, UK

**Keywords:** OverStitch, Endoscopic suturing, Plication, Gastric conduit, Conduit dysfunction, Oesophagectomy

## Abstract

Gastric conduit is the preferred reconstruction following oesophagectomy. Patients can develop delayed gastric conduit emptying because of dilatation and poor drainage. We present a novel technique utilising endoscopic suturing to plicate the gastric conduit, improving drainage, reducing sumping and improving quality of life. An 81-year-old man who underwent an oesophagectomy for pT1bN0M0 oesophageal adenocarcinoma presented 6 years later with nutritional failure, body mass index (BMI) 17.5 and recurrent aspiration pneumonia. The gastric conduit had dilated, resulting in significant redundancy and sumping. Endoscopic suture plication of the conduit was performed to avoid the risks of surgical revision. Expertise developed in the practice of endoscopic sleeve gastroplasty was utilised to perform intrathoracic gastric conduit plication, eliminating sumping and reducing conduit redundancy. Following endoscopic gastric conduit plication, contrast swallow demonstrated improved shape and drainage of the conduit. At 6- and 12-month follow-up the patient reported improved diet tolerance, an increased BMI of 24.7 and there had been no hospitalisations for aspiration. EORTC QLQ OG25 scores improved from 60 to 32. Our novel technique for plication of an intrathoracic gastric conduit represents a useful strategy to increase the longevity and function of gastric conduits in oesophageal cancer survivors and those undergoing benign resections where long-term high-quality function of the conduit is important for optimal patient outcomes.

## Background

Long-term survival following oesophagectomy has improved steadily over the past two decades as a result of advances in neoadjuvant, adjuvant therapies and surgical management.^1^ Surgical resection remains the cornerstone of treatment with curative intent and typically a two- or three-stage approach is used. Reconstruction can be performed using gastric, colonic or jejunal conduits and often gastric conduit pull-up is the preferred reconstruction.

Although the primary concerns after resection are postoperative complications, mortality and oncological outcomes, there is also a significant burden of long-term nutritional problems, which may impact quality of life in long-term survivors. Some of these nutritional problems relate to conduit dysfunction. The conduit often dilates, which impairs peristalsis and allows sumping and the accumulation of food debris. This impairs conduit drainage due to ineffective antral pressures and failure of the gastric pump. The result is delayed gastric conduit emptying (DGCE).^2,3^

Given gradually improving long-term survival rates, there is an increasing population of patients who develop DGCE.^3^ The same problem also affects patients who undergo oesophagectomy for benign indications such as corrosive injury and end-stage achalasia.^4^ This creates a cohort of patients who either are conservatively managed and must accept a poor quality of life with frequent dietetic intervention, or have supplementary feeding in the form of jejunostomy or nasoenteric tube, or in some cases undergo further surgery to revise or replace the gastric conduit with colonic or jejunal interposition.^5–7^

Endoscopic suturing technology has existed in various forms for the past decade and has gradually been developed to the latest products including the OverStitch Sx device (Apollo Endosurgery, Austin, TX, USA) that mounts on a single-channel gastroscope.^7^ The range of procedures utilising endoscopic suturing has expanded and there is now a substantial body of evidence for the use of these techniques for endoscopic sleeve gastroplasty (ESG) for weight loss, transoral outlet reduction after Roux-en-Y gastric bypass, stent fixation and the closure of fistulous defects, leaks or full-thickness resection sites.^8–11^

We present a novel technique based on our experience of ESG, utilising endoscopic suturing to plicate the intrathoracic gastric conduit – endoscopic gastric conduit plication (EGCP).^12,13^ This reduces the calibre of the distal conduit, improving the action of the gastric pump and reducing sumping, both of which result in better conduit drainage, leading to improved nutrition and quality of life. Although this novel technique will probably be suitable for only a minority of patients, it represents a potentially useful method to improve conduit function and longevity in patients refractory to conservative techniques and thereby avoid or defer the need for further major surgery with its inherent risks and protracted recovery.

### Patient selection

Our initial patient was an 81-year-old man who had previously undergone a two-field Ivor Lewis oesophagectomy with pyloroplasty in 2006 for a pT1bN0M0 lower oesophageal adenocarcinoma. He presented in 2021 with nutritional failure, a body mass index (BMI) of 17.5 and recurrent aspiration-induced pneumonia requiring repeated hospital admissions. Computed tomography scanning (CT) with oral contrast and barium swallow showed the gastric conduit had dilated, resulting in significant redundancy and sumping with poor drainage (Figure 1, left). The patient was initially managed with feeding jejunostomy and pyloric dilation. These measures failed to improve BMI or oral intake and conduit drainage, and the patient did not wish to continue with jejunostomy feeding as his only source of nutrition.

**Figure 1:**
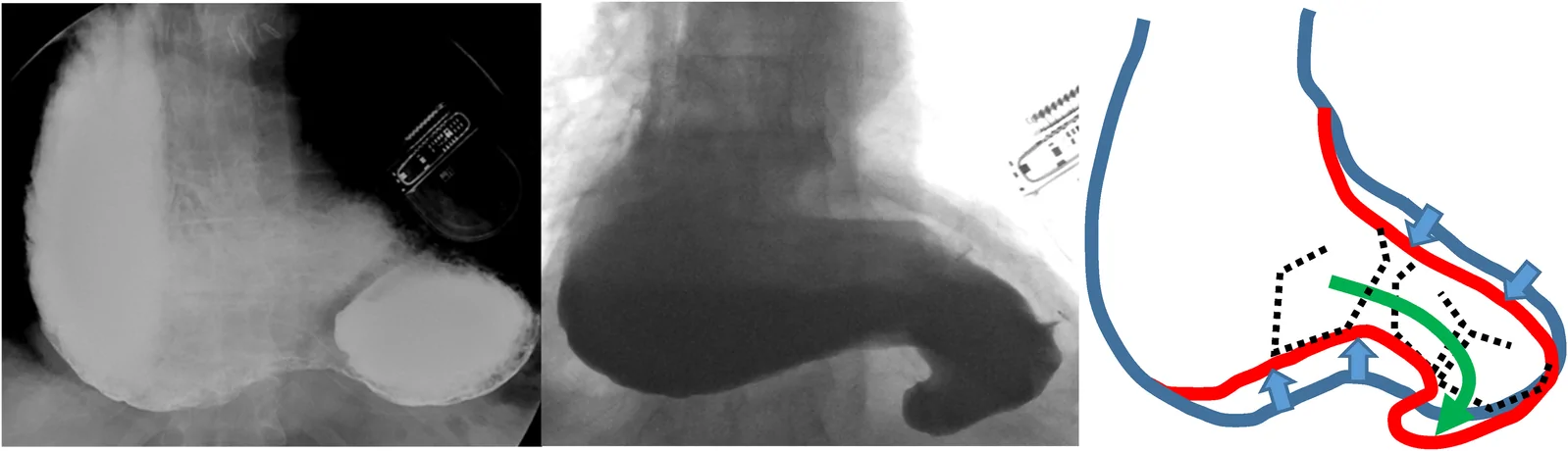
(Left) Pre-procedure barium swallow showing significantly dilated gastric conduit. Of particular functional relevance is the dilated antrum of the distal conduit with sumping and an upward angulation of the pylorus with no contrast drainage to the duodenum. (Middle) Post-procedure water-soluble contrast swallow showing narrowed antrum and downward angulation towards pylorus. Subsequent series showed good drainage to duodenum. (Right) Diagram of sutures and the intended effect. The distal part of the gastric conduit is narrowed using three U-shaped suture patterns to partially plicate the gastric conduit. This improves the action of the antral gastric pump, reduces sumping and causes downward angulation from the antrum to pylorus. Blue line = outline of gastric conduit pre-procedure; red line = outline after procedure; black dashed lines = U-shaped pattern of plication sutures; blue arrows = reduction in diameter of distal conduit; green arrow = downward angulation from antrum to pylorus post procedure.

Endoscopic suture plication of the gastric conduit was therefore discussed with the patient to avoid the risks of surgical revision of the conduit or conversion to colonic interposition.^14^ Following informed consent and a full discussion of the alternatives, the patient chose to undergo the procedure.

## Technique

The patient had a liquid diet for 48h and was fasted for 6h before the procedure. Simethicone solution was given orally prior to the procedure, which was performed under general anaesthesia with endotracheal intubation. Two gastroscopes were used, the first for assessment of the gastric conduit. Copious food debris was found and an oesophageal overtube deployed to allow piecemeal removal of the food using a Roth Net bag. The conduit was irrigated with simethicone solution and carefully aspirated to ensure optimal views. A second gastroscope was then fitted with the OverStitch Sx device manufactured by Apollo Endosurgery (part of Boston Scientific).^7^ A second gastroscope is desirable because the endoscope must be dry prior to fitting of the OverStitch device; however, it is often useful to perform initial diagnostic assessment and washing of the stomach before the final decision to open the OverStitch device and carry out the procedure.

The OverStitch device is connected to the gastroscope using tip mounting of the needle body in a similar manner to a standard gastroscope cap. The needle body is connected to the OverStitch handle via a two-channel tube that runs parallel to the gastroscope and is held with a series of silicone straps. In one channel of the device, an instrument called the Helix is passed. The Helix is a corkscrew-type needle that can be pressed against the tissue and rotated. It drives into the tissue at a rate of 1.1mm per rotation and in the stomach three or four rotations are typically used to penetrate to the submucosa and muscularis propria layer in a careful and calibrated way. This ensures that full-thickness suture bites can be taken, avoiding superficial mucosal bites or bites that are too deep and include organs or structures beyond the stomach. The Helix can then be retracted to draw the tissue into the jaws of the needle body before passage of the needle. Retraction provides further understanding that the stomach wall is free from adjacent organs and allows bites to be taken that are perpendicular to the gastric wall, further ensuring full-thickness bites. The other channel of the OverStitch device is used for the needle, which is mounted on the exchange catheter. The exchange catheter allows the actual needle to be passed back and forth between the needle body and the exchange catheter as bites are taken through the tissue. After all the desired suture bites have been taken the needle is dropped from the exchange catheter and acts as a ‘T’ anchor to secure one end of the suture. The exchange catheter is then removed and the cinch device mounted on the suture and loaded into the same channel. With traction and counter-traction the suture can be tightened before the cinch is deployed and the other end of the suture is locked and then cut. An animation of the technique can be seen in Video 1.

The shape and sumping of the conduit were carefully evaluated to determine the optimal locations for sutures (Video 2). Starting distally in the conduit using the suture placement technique described above, we placed four sutures (Video 3 placing sutures; Video 4 anchor drop, Video 5 cinching), each of which had four to six bites of tissue in a U-shaped orientation across the most dependent and redundant areas of the distal conduit (Figure 1, right). The decision on where to place suture bites was also based on an assessment of the movement of the tissue to ensure no significant adherence to adjacent structures and the safety of the location given the proximity of the heart and pericardium and the need to avoid pericardial or vascular injury. Taking multiple bites with each suture ensured robust and uniform plication of the distal conduit.

### Results

At the end of the procedure, a complete diagnostic gastroscopy was performed to confirm visualisation of the pylorus and passage to the duodenum with ease (Video 6). The total procedure time was 86min. The day following the procedure, the patient underwent a water-soluble contrast swallow that demonstrated improved shape of the conduit (Figure 1, middle). Drainage was also improved, as assessed by the swallow and subsequent plain radiograph. Thereafter, the patient was commenced on a liquid diet and was discharged the following day after dietetic counselling. The total hospital length of stay was 2 days and there were no intra- or postoperative complications. The patient’s diet was progressed over the following four weeks with ongoing dietetic support.

At 6- and 12-month follow-up, the patient reported improved diet tolerance, had an increased BMI of 24.7, and there had been no episodes of hospitalisation. The European Organisation for Research and Treatment of Cancer Quality of Life Questionnaire OG 25 (EORTC QLQ OG25) was utilised to assess patient-related outcomes before and after the EGCP procedure. The EORTC QLQ OG25 asks a series of 25 questions concerning aspects of swallowing, postprandial pain, reflux, nutrition and other quality-of-life parameters. It is based on patient recollection of symptoms in the previous week and patients rate the severity of each symptom on a scale from 1 (not at all) to 4 (all the time). The minimum score is therefore 25 and the maximum is 100. Our patient’s score was 60 before the EGCP procedure, particularly in areas relating to swallowing, postprandial discomfort, early satiety and personal concern regarding health and weight. Following the procedure at 12 months, the patient’s score was 32, with all questions that previously scored 3 or 4 having reduced to 1 or occasionally 2. Some question areas such as dry mouth or difficulty swallowing saliva remained unchanged.

## Discussion

### Patient selection and multidisciplinary assessment

Our technique could represent a useful strategy to increase the longevity and function of the gastric conduit in oesophageal cancer survivors and those undergoing benign resections where long-term, high-quality function of the gastric conduit is important for optimal patient outcomes.

The less-invasive nature of EGCP, when compared with surgery to refashion or replace an intrathoracic conduit, makes it suitable for a wide variety of patients. Potential patients should be evaluated and discussed in a multidisciplinary team including a surgeon, dietician, radiologist and, if needed, a psychologist.

Patients should have had attempts at conservative dietetic optimisation and consideration should be given to the risks and potential benefits of supplemental tube feeding vs an attempt to improve oral nutrition using EGCP. Initial endoscopy and contrast swallow may help to indicate whether treatment should be directed towards the pylorus before or after consideration of EGCP.^15^ Consideration should also be given to either diagnostic/therapeutic Botox to the pylorus, per oral pyloroplasty (POP) or per oral pyloromyotomy (POEM) to assess and improve pyloric drainage if not already performed or deemed inadequate.

### Diagnostic workup

Given the novelty of our technique, there is no standardised pre-procedure diagnostic workup. However, our approach followed a typical clinical evaluation for a DGCE patient. Initially, a staging CT chest abdomen and pelvis and positron emission tomography-CT were performed to exclude tumour recurrence. A contrast swallow and CT scan of the chest and abdomen with oral contrast were performed to evaluate gastric conduit emptying and volume. Lastly, upper gastrointestinal endoscopy was carried out to further assess the anastomosis, conduit shape and volume, the hiatus, and pyloric angulation and tone.

We considered the use of gastric scintigraphy emptying studies and planimetry but felt that the aforementioned investigations were sufficient to establish DGCE and whether the shape of the conduit and location of the sump were suitable for endoscopic suture plication.

### Treatment algorithm

We suggest that EGCP be considered in a step-up treatment algorithm for patients with DGCE. The algorithm should begin with dietetic education, modification and oral nutritional supplementation. If this fails to improve nutrition, BMI and quality of life, then a multidisciplinary discussion should be had with the patient in which the options of nasoenteric or surgically placed enteral supplemental feeding tube are discussed vs treatment options directed at improving conduit function. The relative merits of pyloric drainage procedures (Botox/pyloroplasty/POP or POEM) and/or procedures to modify or replace the conduit, should be assessed in each patient. Those with any suggestion of pyloric-level hold-up may benefit from a diagnostic trial with pyloric Botox injections and, if successful, a pyloric drainage procedure before EGCP.^16,17^ EGCP may then offer the next level in the treatment algorithm, prior to any consideration of surgical conduit replacement or revision. The risks associated with surgical revision or replacement with colonic or jejunal interposition are so significant that EGCP probably represents a safer treatment approach, with the benefits of being repeated as required.^18^

### Technical considerations

We have the largest experience of ESG in UK practice and through this experience and collaborations with others had the following technical considerations to achieve a good result in EGCP.^11,12^

The most important aspect for durability of gastric conduit plication is full-thickness suture bites to ensure that all layers of the stomach, in particular the strength layers of serosa and submucosa, are captured by the suture. The first aspect to ensure this is the accurate use of the Helix device which advances 1.1mm for every full turn of the device. Knowledge of the position in the conduit, (antrum, body, etc) can then inform the appropriate depth to advance the Helix to capture all layers of the stomach without injuring adjacent organs. Of equal importance in ensuring full-thickness suturing without injuring adjacent structures is a trial retraction of the tissue with the Helix. This is to ensure sufficient movement of the gastric wall such that it is unlikely to have any fixed or dense adhesions to adjacent organs, and conversely to demonstrate that the Helix is sufficiently deep in the gastric wall, as shown by the force that can be applied before any pull out occurs. Finally, it is most important to ensure that the jaws of the needle body are aligned strictly perpendicular to the gastric wall to ensure that the needle path crosses all layers of the gastric wall rather than passing obliquely and ‘side swiping’ the mucosa/submucosa.

This year another group has also reported on the use of endoscopic suturing to remodel the shape and function of the oesophagus. The patients in their series with successful outcomes were those with end-stage achalasia. This illustrates another clinical condition in which intrathoracic endoscopic plication can be utilised to improve the drainage and function of the foregut.^19^ This group also report plication of one patient with DGCE but without clinical success. In their report, they preferred to only suture the submucosa. Although this understandably would be sufficient for oesophageal plication in the mega-oesophagus of achalasia, given the strength of the submucosa and lack of serosa, we feel that the lack of full-thickness suture bites when plicating a gastric conduit significantly reduces the strength and durability of the plication.

### Risks

As a novel procedure, the incidence of specific risks is not enumerated. In our case, no complications were observed. However, based on our experience of endoscopic suturing for both ESG and intrathoracic fistula closure, risks include bleeding, perigastric/oesophageal collections, ongoing symptoms/failure and injury to adjacent organs.^8^ However, in our experience and published case series, these risks are low, in the order of 2–3%.^8^ Although the risks might be slightly higher when plicating a gastric conduit in the thoracic cavity, we consider them likely to be substantially lower than with surgical revision or replacement of a dysfunctional conduit. A reduced length of hospital stay is very likely if the existing conduit is preserved and its longevity extended with improved function. Finally, if outcomes are not sufficient after one procedure, the endoscopic plication can be repeated to remove additional redundancy either as a staged procedure or after some time if there is any further dilation or disruption of the sutures.

### Post-procedure evaluation

In the initial use of our EGCP technique, we evaluated the anatomical and functional result with a post-procedure endoscopy, a water-soluble contrast swallow and 12-month clinical follow-up.

In our publicly funded healthcare setting, given that the clinical, nutritional and quality-of-life outcomes for the patient remained excellent, there was no strong reason to perform additional post-procedure investigations.

The change in clinically relevant patient-reported quality-of-life outcomes, as assessed by the EORTC QLQ OG25, provides good evidence of the clinical efficacy of our EGCP procedure. Our patient’s scores reduced from 60 before the procedure to 32 following the procedure. That the post-procedure score of 32 remained low and close to the questionnaire minimum of 25 even at 12 months after the procedure suggests that the procedure is durable in the medium term. Further long-term follow-up will further demonstrate the durability of the procedure. However, the repeatability of the procedure and favourable risk profile mean that even if symptoms recur in the long term, the procedure is still likely to be clinically justified and efficacious.

As our experience with this novel technique grows, we may consider long-term follow-up protocols similar to those used, for example, after POEM. However, given the ease with which an EGCP could be repeated if a patient’s clinical symptoms deteriorated, close radiologic or endoscopic follow-up is probably unnecessary.

### Limitations and future developments

The main limitation with this article on our novel endoscopic procedure is that we report on our early experience. We have begun the diagnostic evaluation of further patients and will continue to refine our technique and gain further experience. Other groups will also hopefully consider our technique and in time we can report on greater case volume and longer-term results.

## Conflicts of interest

Mr Jamie Kelly is a clinical proctor and trainer for Apollo Endosurgery, part of Boston Scientific and receives financial compensation for his time for these activities. Mr Hywel Room, Miss Thakshyanee Bhuvanakrishna, Miss Dimitra Peristeri and Mr Shaun Preston have no conflicts of interest or financial ties to disclose.

## Funding

None.

## Author contributions

HR and TB wrote the manuscript. DP and TB conducted the background literature search to contextualise. JK carried out the procedure assisted by HR and JK. HR conceptualised the article. JK and SP provided a critical review of the manuscript and senior approval of submission.

## Supplementary Information

The online version contains supplementary material available at https://doi.org/10.1308/rcsann.2025.0025.
